# Glutamine antagonist DON attenuates chikungunya virus-induced myositis by suppressing inflammatory activation in a murine model

**DOI:** 10.1080/22221751.2026.2622213

**Published:** 2026-03-06

**Authors:** Xinyu Zhang, Yue Zhang, Jiarui Huang, Zhiyong Ma, Hu Yan, Maohua Zhong, Jingyi Yang, Fengjiao Hu, Mengliu Zeng, Mengji Lu, Huimin Yan, Ejuan Zhang

**Affiliations:** a Department of Infectious Diseases, Zhongnan Hospital of Wuhan University, Wuhan, People’s Republic of China; b Medical Science Research Center, Zhongnan Hospital, Wuhan University, Wuhan, People’s Republic of China; c Mucosal Immunity Research Group, State Key Laboratory of Virology, Wuhan Institute of Virology, Chinese Academy of Sciences, Wuhan, People’s Republic of China; d College of Nursing and Health Management & College of Life Science and Chemistry, Wuhan Donghu College, Wuhan, People’s Republic of China; e Institute of Infection, Immunology and Tumor Microenvironment, Hubei Province Key Laboratory of Occupational Hazard Identification and Control, Medical College, Wuhan University of Science and Technology, Wuhan, People’s Republic of China; f Shanghai Public Health Clinical Center, Fudan University, Shanghai, People’s Republic of China; g Institute for Virology, University Hospital of Essen, University of Duisburg-Essen, Essen, Germany

**Keywords:** Chikungunya virus, glutaminolysis, metabolic modulation, 6-diazo-5-oxo-L-norleucine, T cell responses, immunopathology

## Abstract

Chikungunya virus (CHIKV), an emerging mosquito-borne alphavirus, induces debilitating polyarthralgia and myositis with no licensed specific therapeutic drugs. This study investigates the virological, immunological, and pathological consequences of targeting glycolysis and glutaminolysis during CHIKV infection. *In vitro*, either glucose/glutamine deprivation, or pharmacological inhibition by 2DG/DON significantly suppressed viral replication in mammalian cell lines. *In vivo*, however, differential tissue biodistribution dictated that neither inhibitor reduced viral loads in serum or foot tissues of acute infected mice following footpad inoculation with 10⁴ PFU CHIKV. Strikingly, DON, but not 2DG, abolished histopathological manifestations of myositis and inflammatory infiltration despite comparable viral burdens. Mechanistically, DON-mediated tissue protection was related to dual immunomodulation. DON significantly depleted splenic innate immune cells, including monocytes and macrophages, which play roles in driving tissue inflammatory infiltration cascades. Meanwhile, DON inhibited CD4 + and CD8+ T cell effector programmes, resulting in suppressed activation marker (CD44) expression and effector cell differentiation (decreased effector: naive ratio and TEM: TCM balance). The proliferative capacity (Ki-67 + cells), polyfunctional cytokine responses (IFN-γ+, TNF-α and IL-17 + cells) and cytotoxicity potential (CD107a + cells) of CD4 + and CD8+ T cells were significantly impaired by DON injection. Crucially, glutaminolysis inhibition uncoupled immunopathology from viral containment, attenuating tissue damaging immunity while preserving baseline antiviral competence. Collectively, these findings establish host glutamine metabolism as a pharmacologically tractable target for alphavirus-induced arthritis, demonstrating that selective immunometabolic modulation resolves the severe acute inflammatory pathology.

## Introduction

Chikungunya virus (CHIKV), a mosquito-borne alphavirus, induces severe polyarthralgia and myositis in over 75% of infected individuals, with 30–60% developing persistent or recurrent joint pain lasting months to years [1,2]. In the past decades, CHIKV has caused outbreaks or isolated cases worldwide, including Europe, Asia, Africa, and the Americas [3,4]. However, there are no licensed specific antiviral drugs or targeted therapies available for clinical use [5,6]. Several new approved vaccines require the clinical evaluation of prophylactic efficiency [7,8]. Current treatment relies on analgesics and non-steroidal anti-inflammatory drugs [9,10], highlighting an urgent need to develope mechanism-based intervention approaches and agents.

CHIKV viremia typically resolves within 1–2 weeks, with acute symptoms subsiding between days to weeks after viremia clearance [11,12]. Viral elimination requires coordinated innate and adaptive immune responses mediated by intradermal γδ-T cells, macrophages, NK cells, and T/B lymphocytes, while these anti-viral immune cells play an important role in CHIKV-induced diseases [13,14]. Histopathological analysis reveals characteristic tissue damage marked by myocyte necrosis, synovial hyperplasia along with inflammatory infiltration in musculoskeletal tissues and articular cavities [15,16]. Mechanistic studies establish macrophages and CD4+ T cells as central pathological mediators [17–19], with granzyme A identified as their key cytolytic effector in triggering the CHIKV-induced immunopathology [20,21]. Targeted depletion of these components in murine models significantly attenuates foot swelling and tissue damage without impairing viral clearance [18,22,23]. In a mouse model of chronic CHIKV infection, virus-induced antiviral CD8+ T cell responses and antibodies failed to clear the virus or prevent chronic arthritis development [24]. These findings indicate that CHIKV triggers an excessive immune activation that is not essential for viral clearance but mediates tissue damages. Therefore, targeted immunomodulation could be a therapeutic strategy to ameliorate CHIKV-induced diseases.

Cellular energy metabolism critically regulates immune cell activation and function. Antiviral immune responses require metabolic reprogramming to sustain effector cell activation and functions [25,26]. Inhibition of glycolytic or glutaminolytic fluxes impairs antiviral immunity by suppressing T cell accumulation, proliferation, activation, and cytokine production [27,28]. The glucose analog 2-deoxy-D-glucose (2DG) and glutamine antagonist 6-diazo-5-oxo-L-norleucine (DON) ameliorate immunopathology in COVID-19, HSV and Sindbis infection models by limiting inflammatory cell recruitment and cytotoxicity [29–32]. However, these therapeutic benefits entail risks since metabolic suppression may delay viral clearance by compromising immune responses [33]. Nevertheless, parallel assessment of these inhibitors remains limited, hindering identification of the optimal metabolic blockade treatment for acute self-limited infections. As a result, comparative evaluation in CHIKV infection models is needed to determine whether glycolytic or glutaminolyitc modulation could achieve immunopathology reduction without exacerbating viral control.

In the present study, we systematically evaluated and compared the therapeutic efficacy and mechanisms of 2DG and DON in CHIKV-infected murine models. Both inhibitors effectively suppressed viral replication *in vitro*, while only DON alleviated myositis and joint damage *in vivo*. This therapeutic dichotomy is related to suppressed CD8+ T cell responses with minimal impact on viral replication, indicating that glutaminolysis inhibition preferentially targets immunopathological pathways. This metabolic specificity provides a strategic advantage over broad-spectrum immunosuppression. Our findings position host metabolism as a pharmacologically tractable target for alphavirus-induced arthritis in the CHIKV infected mouse model.

## Materials and methods

### Mice

Female C57BL/6 mice (6–8 weeks) were used to study virology and immunopathogenesis following CHIKV infection [34–36]. Mice were purchased from Beijing Vital River Laboratory Animal Technology (Beijing, China). Animals were housed in level-3 isolators (Isocage, Tecniplast, Italy) within an animal biosafety level 3 (ABSL-3) facility at the Wuhan Institute of Virology, Chinese Academy of Sciences (WIV, CAS), following the Guidelines for Animal Care and Use of WIV and Regulations for the Administration of Affairs Concerning Experimental Animals in China (1988). All procedures were conducted in ABSL-3 biological safety cabinets under protocols approved by the Ethics and Biosafety Committees and the Institutional Review Board of WIV, CAS (No. WIVA09201706).

### Virus

The CHIKV strain (KC488650, Asian lineage) was obtained from the National Virus Resource Center, WIV, CAS. Strain KC488650 originated from a CHIKV-positive patient in China and is phylogenetically related to strain 0706aTw isolated in Indonesia in 2007, as reported previously [37]. All experiments involving live viruses were performed in a BSL-3 laboratory.

### Cell culture and nutrition deprivation assay

The BHK-21 cell line was cultured in Dulbecco’s Modified Eagle Medium (DMEM, Gibco) supplemented with 10% fetal bovine serum (FBS, Gibco) and 1% penicillin/streptomycin at 37 ℃ in a 5% CO₂ atmosphere. For nutrition deprivation assays, cells were cultured with either glucose-free DMEM or glutamine-free DMEM supplemented with 10% dialyzed FBS (Gibco, A3382001). Control groups received complete DMEM. Parallel pharmacological interventions employed 2 mM 2DG (Sigma, D8375) to inhibit glycolysis or 1 μM DON (Sigma, D2141) to suppress glutaminolysis, with PBS as vehicle control. CHIKV was diluted with the respective deprivation or inhibitor-containing medium. Cells were then infected with diluted viruses at MOI = 1 and maintained in corresponding conditional mediums for 24 h. Viral replication was quantified at 24 h post-infection.

### Mouse infection and sampling

C57BL/6 mice (6–8 weeks old) were intradermally inoculated in the left hindlimb footpad with 10 μl of PBS diluted CHIKV containing 10^4^ PFU viruses. Control mice received PBS-diluted supernatant from mock-infected BHK-21 cells. Foot swelling was quantified using a digital calipre by measuring both the height (thickness) and breadth of the inoculated footpad. The degree of swelling was determined as the percentage increase in the size of footpad (height × breadth) over the preinfection baseline, using the formula: [(size at day x − size at day 0) / size at day 0] × 100%. Mice were sacrificed at 7 days post-infection (dpi) for virological and histopathological analysis (n = 6–7 per group).

For metabolic intervention in infected mice, 2DG (1 g/kg) [38,39] or DON (0.3 mg/kg) [40,41] were diluted in PBS and delivered via intraperitoneal injection in 200 ul volume. Inhibitors were administered 0.5 h prior to viral challenge and continued daily for subsequent 4 days; mice were then sacrificed at 7 dpi. For metabolic intervention in uninfected mice, animals received a single dose of inhibitors and then sacrificed at 24 h post-injection.

For viral quantification, tissue samples were weighed and homogenized twice at 5000 rpm for 20 s by a Tissue Cell destroyer D1000 (NZK Ltd., Wuhan, China) in 500 μl diluent (DMEM supplemented with 2% FBS) per 100 mg tissue.

### Histopathology examinations

Foot tissues were obtained from at least three mice per group at 7 dpi, and fixed in 10% neutral-buffered formalin for ≥48 h. Fixed tissues were decalcified with EDTA solution for 15 days, and stored in 70% ethanol. Samples were embedded in paraffin, sectioned longitudinally at 5 μm, and stained with hematoxylin and eosin (H&E).

### Plaque assay

Viral titres of stock solutions or infected cell supernatants were quantified by plaque assay in BHK-21 cells as described previously [42]. Briefly, 10-fold serial dilutions of viral samples were added to duplicate cell monolayers of BHK-21 cells. After virus absorption for 1 h, cells were overlayed with 1.25% methyl cellulose medium (Sigma–Aldrich, USA). Cells were incubated for 48 h, fixed with 4% paraformaldehyde-PBS for 30 min, and stained with 1% crystal violet for 4 h. Plates were rinsed extensively with deionized water to visualize and calculate plaques.

### Quantitative RT–PCR detection of viral RNA

Total RNA was extracted from 2 × 10^6^ cells or 50–100 μl tissue homogenate. For total viral RNA and strand-specific RNA detection, cDNA synthesis and qPCR were performed as previously described [24]. For host genes detection, cDNA was synthesized and qPCR were performed as previously described [28]. CHIKV RNA was quantified using standard curves, while host gene expression was normalized to saline controls.

### Energetic metabolic profiling of T cells

Metabolic profiling of splenic immune cells was performed using the SCENITH assay, which infers ATP production capacity by measuring puromycin incorporation into newly synthesized proteins [43]. Briefly, splenic single-cell suspensions were treated for 30 min with metabolic inhibitors, including 2DG (100 mM; a glycolysis inhibitor), Oligomycin (1 μM; an oxidative phosphorylation inhibitor), or a combination of both. Puromycin (10 μg/ml, Sigma) was added for the final 20 min of inhibitor treatment. Cells were then washed with cold PBS and FC block (Biolegend) for 15 min. Cell surface was stained at 4 ℃ for 30 min and fixed with Fixation/Permeabilization Solution Kit (BD Biosciences). Intracellular staining with an anti-puromycin antibody (clone 2A4, Biolegend) was performed in perm-buffer at 4 ℃ for 1 h. The geometric mean fluorescence intensity (GeoMFI) of the anti-puromycin signal was quantified by flow cytometry for each condition. Metabolic dependency profiles, including glucose dependence, FAAO capacity, mitochondrial dependence and glycolytic capacity, were calculated based on the translation rates (GeoMFI of anti-puromycin) under the respective inhibitor treatments.

### Analysis of T cell activation and function

Splenocyte suspensions were prepared by homogenization as described previously [28]. To evaluate CHIKV E2 protein-specific T cell responses, splenocytes were stimulated with purified E2 protein at 2 μg/ml for 72 h. IFN-γ production was quantified using a commercial ELISPOT assay kit (eBioscience, USA) following manufacturer instructions. The spots enumeration was counted manually, and E2-specific CD8+ T cell responses were calculated by subtracting background values obtained from negative controls stimulated by HIV-derived P24 protein.

Splenocytes were measured by direct staining, or intracellular cytokine staining after activating with PMA (200 ng/ml) and ionomycin (10 μg/ml) for 4.5 h, as described previously [24]. Cell surface staining was performed using eBioscience or Biolegend reagents, including anti-CD4 (clone L3T4), anti-CD8 (clone 53–6.7), anti-CD11b (clone M1/70), MHC-II (clone M5/114.15.2), anti-CD19 (clone 6D5), anti-CD62L (clone MEL-14), anti-CD44 (clone IM7), and anti-CD127 (clone A7R34) antibodies. Dead cells were excluded by Fixable Viability Dye (FVD, eBioscience, USA). Subsequent intracellular staining utilized the Cytofix/Cytoperm kit (eBioscience, USA) with antibodies against IFN-γ (clone XMG1.2), TNF-α (clone MP6-XT22), IL-17 (clone TC11-18H10.1) and CD107a (clone 1D4B). Cell proliferation was detected by fixation with the Nuclea Transcription Factor Buffer Set followed by intranuclear staining with anti-Ki-67 (clone 16A8). Stained cells were acquired on an LSRFortessa flow cytometer (BD Bioscience, USA) with data analysis using FlowJo software (Tree Star, Ashland, OR). Gating strategy was shown in Fig. S1A. Cytokine producing CD4 + and CD8+ T cells were calculated by subtracting background values from the unstimulated control. Details of all reagents and instruments used in the experiments were summarized in Table S1.

### Statistical analysis

Statistical analysis was performed using GraphPad Prism software. Statistical differences were analyzed by unpaired Student t-test, one-way ANOVA or two-way ANOVA tests. The *p*-values < 0.05 were considered significant (* indicated *p* *<* 0.05; ** indicated *p* *<* 0.01; *** indicated *p* *<* 0.001; ns, not significant). All data points are representative of two independent experiments.

## Results

### Experimental CHIKV infection induces virological and immunological changes in adult mice

Our previous studies established a neonatal mouse model exhibiting typical acute and recurrent chronic histopathology progression following infection with CHIKV strain KC488650 [24]. To establish viral infection in mice with developed immune system, adult C57BL/6 mice were infected by intradermal inoculation in the footpad with 10^4^ PFU of CHIKV strain KC488650. Infection with this dosage results in an acute viral infection and tissue injury in mice.

**Figure 1.**
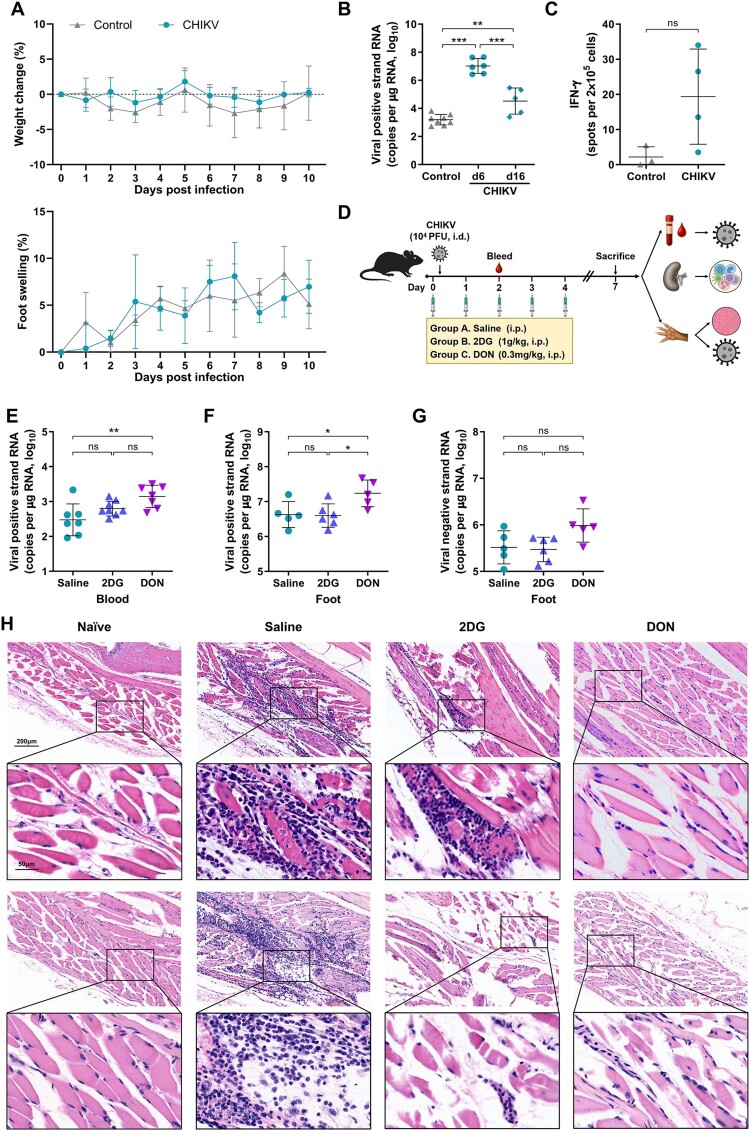
Pathological and virological profiles in CHIKV-infected mice treated with metabolic inhibitors. Adult C57BL/6 mice were infected intradermally with 10^4^ PFU CHIKV, with control mice receiving BHK21 cell supernatant. (A) Body weight changes and foot swelling kinetics were monitored post-infection. (B) Viral positive-strand RNA levels in inoculated foot tissues were quantified by qRT-PCR at 6, and 16 dpi. (C) E2-specific T cell responses were assessed by ELISPOT at 16 dpi following 72 h stimulation with purified E2 protein. (D) Schematic diagram of the experimental design. (E-F) Viral positive-strand RNA levels in blood or foot tissues were measured at 2 dpi or 7 dpi, respectively. (G) Viral negative-strand RNA levels in foot tissues were measured at 7 dpi. (H) H&E-stained foot tissue sections were examined at 7 dpi. Histological data were representative of at least 3 mice per group, with two individual sections shown. All qRT-PCR analysis used nsP1-specific primers and probes. Data represented mean ± SD. n = 5−6 mice per group. Statistical significance was determined by two-way ANOVA (A), one-way ANOVA (B, E-G) or student’s t test (C). *: *p* *<* 0.05; **: *p* < 0.01; ***: *p* < 0.001.

Consistent with our previous reports [24], adult mice infected with strain KC488650 exhibited neither measurable weight loss nor foot swelling (Figure 1A), while the viral genomic RNA and replication intermediates were present in tissues of all individuals. Viral replication in foot tissue peaked at around 6−7 dpi and rapidly decline to a low level by 16 dpi, with viral clearance achieved in approximately 40% of mice (Figure 1B). CHIKV induced predominantly Th1-type immune response in adult mice (Fig S1B-C). Virus-specific IFN-γ producing T cells reached detectable level in most mice at 16 dpi, indicating an established viral specific adaptive immune response (Figure 1C). All infected mice developed prototypical acute pathology characterized by multifocal myofiber necrosis, dense inflammatory infiltrates, and interstitial edema (Figure 1H, saline panel). Significant pathological changes were restricted to the musculature of injected feet, but rarely involved the joints, knees, or the contralateral feet. These data collectively indicate that CHIKV establishes an effective acute infection and successfully activates the host adaptive immune response in adult mice.

### Glutaminolysis inhibition abrogates CHIKV-induced immunopathology independent of viral burden

To investigate the therapeutic potential by metabolic modulation in CHIKV infected cells, metabolic reprogramming during infection was evaluated in mammalian cell line. Transcriptional upregulation of glycolytic enzymes (*Ldha*, *Eno1*) and glutaminolytic regulators (*CD98*, *Gls2*) were observed in BHK21 cells at 24 h post-infection. (Fig. S2A, S2B), indicating virus-induced activation of glucose and glutamine metabolic pathways. Targeted disruption through glucose or glutamine deprivation, as well as pharmacological inhibitors using 2DG or DON, significantly suppressed viral replication *in vitro* (Fig. S2C-J).

To assess therapeutic efficacy *in vivo*, 2DG and DON were administered intraperitoneally for five consecutive days during CHIKV infection in adult mice (Figure 1D). A transient body weight loss and reduced activity were observed during DON administration but rapidly resolved after treatment cessation. Virological and histopathological parameters were analyzed at 7 dpi, coinciding with peak viral replication and inflammatory pathology. Unlike the direct antiviral effects observed *in vitro*, systemic metabolic modulation exhibited limited impact on viral replication. 2DG administration showed no significant effect on viral RNA in serum (Figure 1E) or foot tissue (Figure 1F). DON treatment resulted in modest elevations of genomic RNA in serum (Figure 1E) and foot tissue (Figure 1F), while viral replication intermediates remained unchanged (Figure 1G). These observations indicate that metabolic intervention does not substantially suppress CHIKV replication *in vivo*.

Histopathological analysis revealed distinct therapeutic outcomes between treatment groups. Compared to saline-treated controls, 2DG treatment partially attenuated tissue damage with reduced inflammatory infiltrates and necrosis. DON administration induced near-complete resolution of immunopathology, characterized by eliminated inflammatory infiltration, preserved muscle architecture, and only sporadic degeneration of isolated skeletal muscle cells (Figure 1H). Remarkably, these protective effects occurred despite sustained viral RNA levels in DON-treated animals, demonstrating effective uncoupling of immune-mediated pathology from viral replication.

### Systemic metabolic inhibitors mediate tissue-selective immunomodulation in naive mice

**Figure 2.**
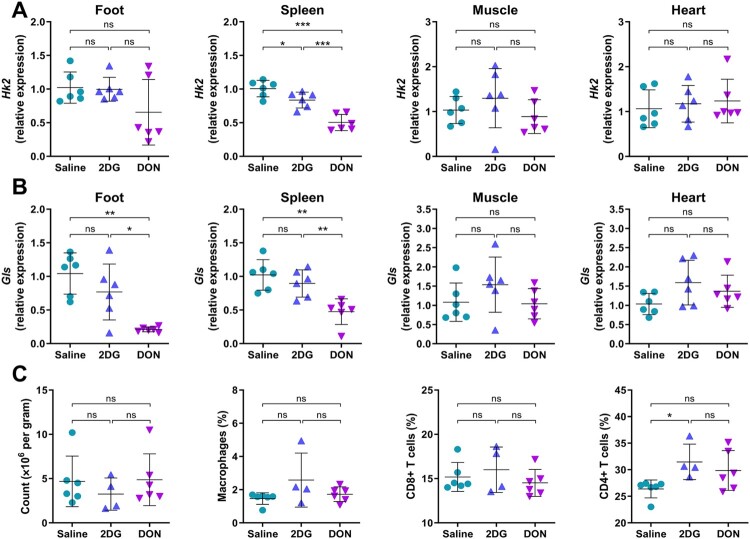
Tissue-Selective metabolic modulation by systemic administration of 2DG and DON. C57BL/6 mice received a single intraperitoneal injection of 2DG (1 g/kg), DON (0.3 mg/kg) or saline. Tissues from footpad, spleen, heart and hindlimbmuscle were harvested 24 h post-treatment. (A) *Hk2* and (B) *Gls* mRNA expression in tissue homogenates were quantified by qRT-PCR. Relative gene expression was normalized to β-actin and calculated relative to saline treated controls. (C) Splenic cellularity and immune cell subset frequencies including macrophages, CD4 + and CD8+ T cells were analyzed by flow cytometry. Data represented mean ± SD. n = 5−6 mice per group. Statistical significance was determined by one-way ANOVA. *: *p* < 0.05; **: *p* < 0.01; ***: *p* < 0.001.

To elucidate the mechanistic basis for the differential antiviral efficacy of 2DG and DON *in vitro* and *in vivo*, tissue-specific analysis of metabolic target engagement was performed in naive mice following a single-dose administration of 2DG or DON. As the primary molecular target of 2DG, *Hk2* mRNA expression was moderately reduced in spleen but unchanged in other tissues after 2DG administration (Figure 2A). Similarly, glutaminase (*Gls*) expression, the molecule target of DON, was markedly downregulated in spleen, while remaining unchanged in neither skeletal muscle nor heart tissues despite significant reduction in foot tissues (Figure 2B). Moreover, DON treatment significantly reduced the expression of chemokines *Ccl3* and *Ccl5*, with minimal change in *Ifnb* and *Isg15* in foot tissue (Fig. S3A, S3B), indicating an impaired chemotactic ability to recruit inflammatory cells alongside a preserved innate antiviral activity. These findings demonstrate preferential modulation of lymphoid organs over musculoskeletal tissues by systemic administration of metabolic inhibitors.

**Figure 3.**
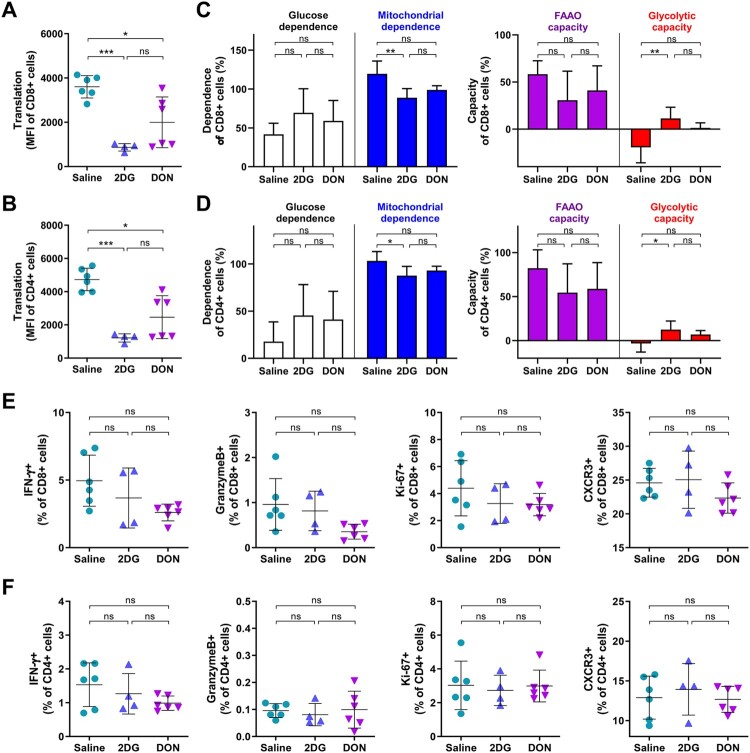
Mild metabolic and functional alterations in T cells from naive mice treated with 2DG or DON. C57BL/6 mice received a single intraperitoneal injection of 2DG (1 g/kg), DON (0.3 mg/kg) or saline. Splenic T cells were isolated 24 h post-treatment. (A) Puromycin incorporation by CD8 + and (B) CD4+ T cells were analyzed by Geomean fluorescence of anti-puromycin. (C) Metabolic profile of of CD8+ T cells and (D) CD4+ T cells were analyzed by SCENITH. (E) Function profiles of CD8+ T cells and (F) CD4+ T cells were assessed by flow cytometry for IFN-γ, Granzyme B, Ki-67 and CXCR3 expression. Data represented mean ± SD. n = 5−6 mice per group. Statistical significance was determined by one-way ANOVA. *: *p* < 0.05; **: *p* < 0.01; ***: *p* < 0.001.

Further characterization revealed that unchanged splenic lymphocyte composition masked profound metabolic reprogramming (Figure 2C). Scenith analysis via puromycin incorporation assay showed that 2DG concurrently suppressed protein synthesis, as well as increased glycolytic capacity and reduced mitochondrial oxidative metabolism in CD8 + and CD4+ T lymphocytes (Figure 3A, B). DON induced mild metabolic perturbation (Figure 3A, B). These metabolic alterations corresponded to functional impairment of immune cells, evidenced by mild reduction of frequencies of IFN-γ+ and Granzyme B + CD8+ T cells (Figure 3C). Proliferation, function and mobility were not significantly affected in CD4+ T cells (Figure 3D). Collectively, systemic metabolic inhibitors primarily exert immunosuppressive effects on splenic immune cells rather than direct antiviral activities at viral replication sites.

### Glutaminolysis inhibition selectively remodels splenic immune cell composition during CHIKV infection

**Figure 4.**
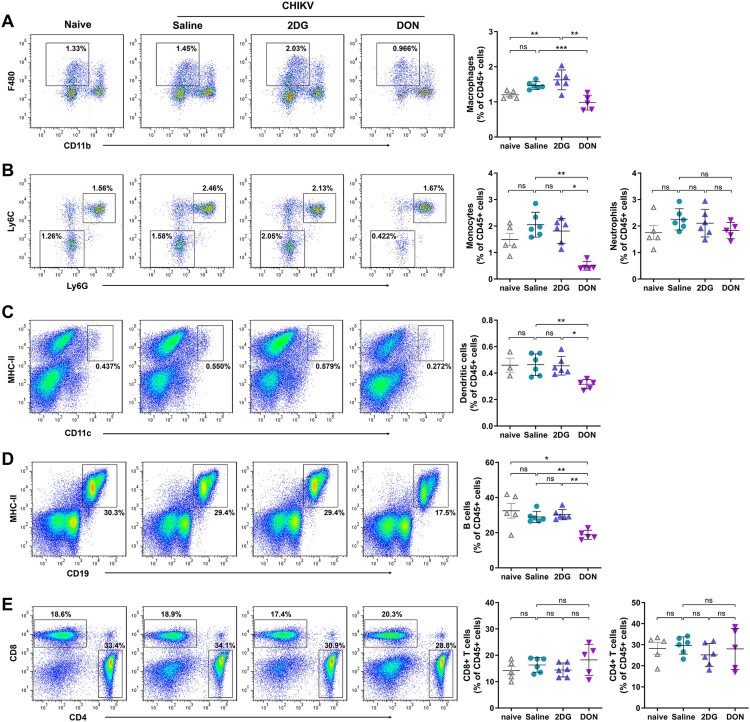
Splenic immune cell composition in CHIKV-infected mice treated with metabolic inhibitors. C57BL/6 mice received intraperitoneal injections of 2DG (1 g/kg), DON (0.3 mg/kg) or saline for 5 consecutive days starting at infection. Freshly isolated splenocytes were analyzed by flow cytometry at 7 dpi. (A) Frequencies of macrophages, (B) monocytes, neutrophils, (C) DCs, (D) B cells, (E) CD4+ T cells and CD8+ T cells were measured. Three naive mice were included as baseline controls. Data represented mean ± SD. n = 5−6 mice per group. Statistical significance was determined by one-way ANOVA. *: *p* < 0.05; **: *p* < 0.01; ***: *p* < 0.001.

Given the critical role of glutaminolysis in lymphocyte proliferation and activation, DON-mediated immunomodulatory effects on splenic immune cells were analyzed during acute myositis at 7 dpi. CHIKV infection induced selective expansion of splenic myeloid lineages relative to naive mice, with elevated frequencies of macrophages (Figure 4A), monocytes (Figure 4B), and neutrophils (Figure 4B).

In the CHIKV-infected mice, 2DG treatment did not significantly alter these infection-induced immune cell frequencies compared to the saline-treated controls. In contrast, DON administration induced selective-immunosuppression within both innate and adaptive immune compartments. Frequencies of macrophages (Figure 4A), monocytes (Figure 4B), DCs (Figure 4C), and B cells (Figure 4D) were all reduced in comparison to saline-treated controls, and even decreased to below the baseline levels observed in naive mice. This cell-type-specific hyper-suppression was most pronounced in macrophages (Figure 4A) and monocytes (Figure 4B), which function as key initiators of inflammatory cell recruitment in CHIKV pathology. Notably, T cells remained refractory to DON-mediated numerical depletion, with CD8 + and CD4+ T cell frequencies matching those in saline-treated controls (Figure 4E), suggesting qualitative rather than quantitative immunomodulation on T cells.

### DON suppresses virus-induced T cell activation and effector differentiation

**Figure 5.**
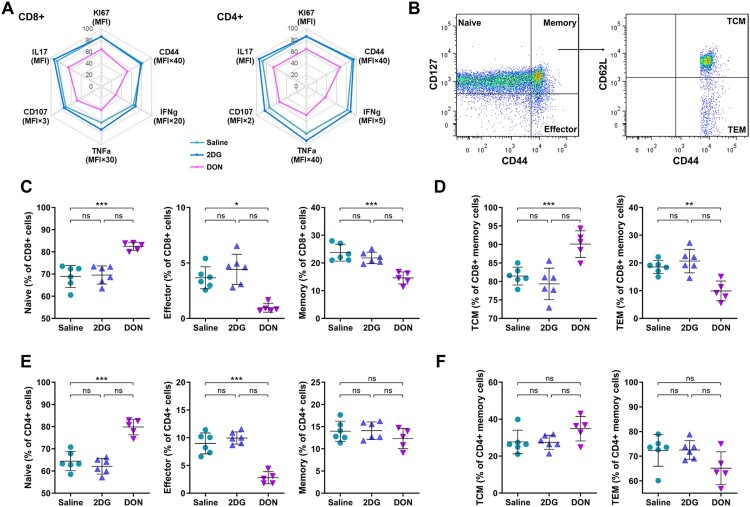
Suppression of viral-induced T cell activation and differentiation via glutaminolysis blockade during CHIKV infection. C57BL/6 mice received intraperitoneal injections of 2DG (1 g/kg), DON (0.3 mg/kg) or saline for 5 consecutive days. Freshly isolated splenocytes were analyzed by flow cytometry at 7 dpi. (A) Activation (CD44), proliferation (Ki-67), cytotoxicity (CD107a), and cytokine production (IFN-γ, TNF-α and IL-17) in CD4 + and CD8+ T cells were assessed by MFI. (B) Gating strategy of T cell differentiation. (C-F) Frequencies of naive, effector, memory, TCM, and TEM populations in CD4 + and CD8+ T cells were quantified. Data represented mean ± SD. n = 5−6 mice per group. Statistical significance was determined by one-way ANOVA. *: *p* < 0.05; **: *p* < 0.01; ***: *p* < 0.001.

In light of the DON-mediated depletion of antigen-presenting cells, such as macrophages, monocytes, DCs, and B cells, the functional consequences for T cell activation were systematically evaluated in CHIKV-infected animals. Flow cytometric analysis of unstimulated splenocytes at 7 dpi revealed broad attenuation of virus-driven T cell activation markers in the DON-treated group but not the 2DG-treated group. CD44 expression, a hallmark of antigen-experienced T cells, was markedly reduced on both CD8 + and CD4+ T cells from DON-treated mice compared to saline-treated controls (Figure 5A). Constitutive expression of effector cytokines (IFN-γ, TNF-α), cytotoxic markers (CD107a) and proliferative marker (Ki-67) showed parallel declines in both T cell subsets following DON treatment. Decreased mean fluorescence intensity (MFI) of these molecules confirmed DON suppression at the single-cell level.

DON administration fundamentally reshaped CD8+ T cell differentiation landscapes. Effector (CD127-CD44+) and memory (CD127 + CD44+) populations were drastically contracted, while naïve (CD127 + CD44-) phenotype became predominant (Figure 5B-D). Memory subset analysis demonstrated preferential loss of effector memory T cells (TEM, CD62L^lo^), with central memory T cells (TCM, CD62L^hi^) emerging as the dominant population. CD4+ T cells displayed analogous but attenuated differentiation arrest, with preserved memory pool integrity despite effector subset reduction (Figure 5E-F). Collectively, these data demonstrate that DON-mediated glutaminolysis inhibition suppresses virus-induced T cell activation and effector differentiation, thereby ameliorating immunopathology while compromising antiviral T cell immunity.

### Glutaminolysis restriction attenuates polyfunctional T cell responses

To evaluate the functional potential of antiviral T cells during acute infection, splenocytes were stimulated with PMA/ionomycin to assess effector capacity independent of antigen specificity. Functional profiling revealed preserved T cell responsiveness in 2DG-treated mice but broad impairment in DON-treated animals across both CD4 + and CD8+ T cell compartments.

**Figure 6.**
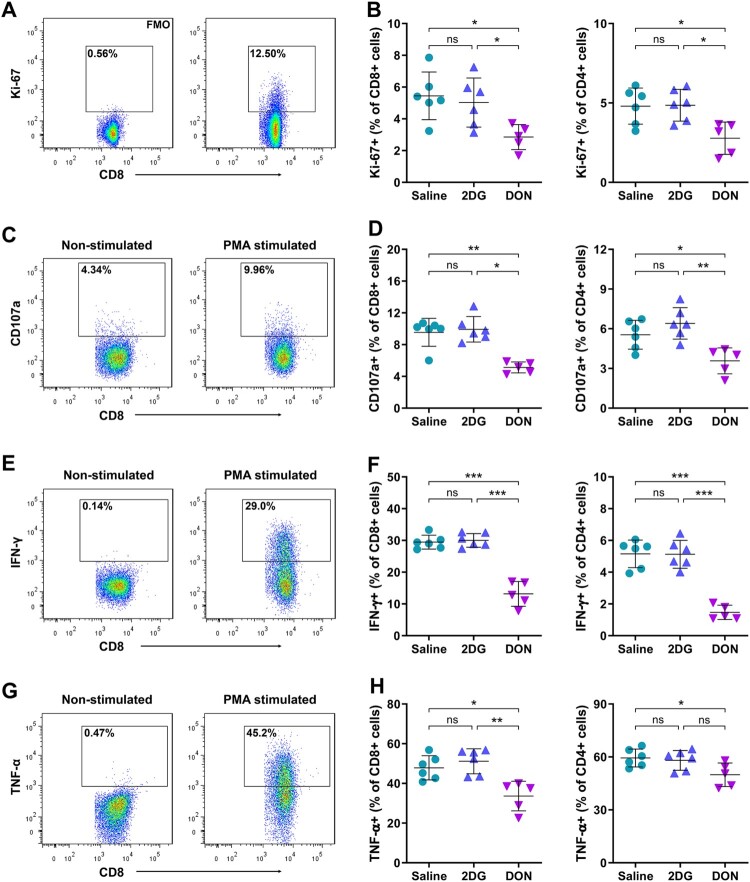
DON-induced impairment of T cell proliferation and antiviral functions in CHIKV infection. C57BL/6 mice were infected with CHIKV and treated with 2DG (1 g/kg), or DON (0.3 mg/kg) for 5 continuous days, splenocytes were isolated at 7 dpi and stimulated with PMA/Ionomycine for 4.5 h before intracellular staining. (A, B) Proliferation (Ki-67+), (C, D) cytotoxicity (CD107a+), (E, F) IFN-γ production and (G, H) TNF-α production by CD4 + and CD8+ T cells were analyzed. Data represented mean ± SD. n = 5−6 mice per group. Statistical significance was determined by one-way ANOVA. *: *p* < 0.05; **: *p* < 0.01; ***: *p* < 0.001.

**Figure 7.**
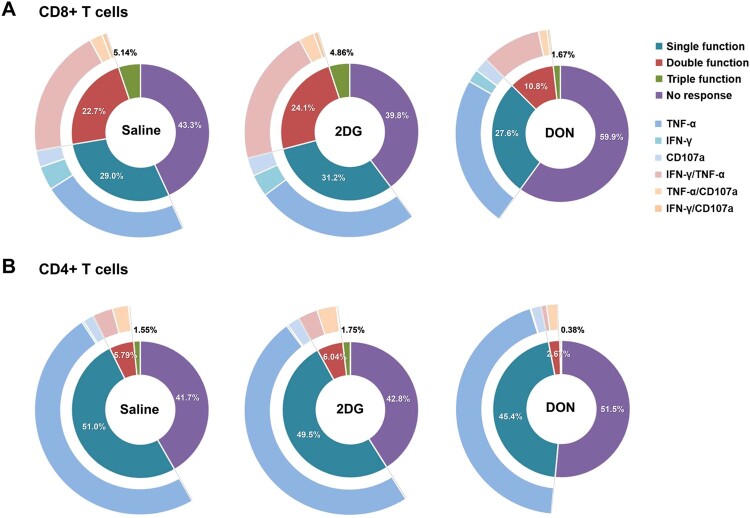
DON-mediated attenuation of polyfunctional T cell responses in CHIKV infection. C57BL/6 mice were infected with CHIKV and treated with 2DG (1 g/kg), or DON (0.3 mg/kg) for 5 continuous days. Splenocytes were analyzed at 7 dpi after stimulation with PMA/Ionomycin. (A) Combinatorial cytokines and cytotoxicity profiles in CD8 + and (B) CD4+ T cells. Pie charts depict proportions of cells producing the indicated combinations of IFN-γ, TNF-α, and CD107a.

DON substantially reduced T cell proliferative capacity, evidenced by diminished Ki-67 + populations (Figure 6A-B). Cytotoxic potential, measured through CD107a surface mobilization (Figure 6C-D), and cytokine production (IFN-γ, TNF-α) were comparably attenuated, indicating significant inhibition of effector functions (Figure 6E-H). Functional phenotyping further demonstrated partial erosion of response complexity. Modest reductions in single-positive populations (CD107+, IFN-γ+, or TNF-α+) contrasted with significant depletion of dual-positive subsets (CD107 + IFN-γ+, CD107 + TNF-α+, IFN-γ+TNF-α+), while triple-positive (CD107 + IFN-γ+TNF-α+) cells were nearly eliminated (Figure 7A-B). This hierarchical functional impairment aligns with the principle that polyfunctional T cell responses are critical for optimal viral clearance. However, the partial retention of monofunctional T cell activity sustains baseline antiviral capacity.

## Discussion

This study systematically characterizes the therapeutic potential of metabolic interventions for balancing viral control and tissue protection during CHIKV infection. Our findings demonstrate that while glycolytic and glutaminolytic inhibition comparably suppress viral replication *in vitro*, only glutaminolysis antagonism via DON confers significant tissue protection *in vivo*. Notably, DON administration induced near-complete resolution of myositis despite minimal alterations in systemic viral loads, effectively uncoupling inflammatory pathology from viral burden. The protective efficacy of DON directly correlates with its immunosuppressive properties, which paradoxically transform a historically detrimental effect into a therapeutic advantage in alphavirus pathogenesis. These results establish glutamine metabolism as a potential target for immunomodulation in acute alphavirus infections.

Host metabolic pathways fundamentally regulate viral replication by supplying energy and biosynthetic precursors, demonstrated in CHIKV and conserved across diverse viruses including hepatitis B virus, dengue virus, Sindbis virus, norovirus, and HIV [44–46]. However, neither 2DG nor DON administration suppressed CHIKV replication in peripheral tissues *in vivo*, presumably because of limited biodistribution to infection sites, as indicated by unaltered expression of molecular targets *Hk2* and *Gls2* in muscle and foot tissues. Conversely, both inhibitors, especially DON, profoundly remodelled splenic immune cell composition and function in CHIKV-infected mice. This differential sensitivity aligns with the established metabolic dependence of lymphocytes on glucose and glutamine for activation and proliferation [47–49].

Immune cells exhibit dual roles in mediating viral control and driving immunopathology during CHIKV infection [10,12,50]. This study demonstrates that DON-mediated glutaminolysis inhibition achieves therapeutic resolution of pathology induced by over-activated immune responses rather than incontrollable viral replication. Specifically, DON depletes pathology-initiating myeloid populations, including macrophages and monocytes, and restricts activation and effector differentiation of both CD4 + and CD8+ T cells. Th17 cells, which contribute to CD4+ T cell-mediated pathology, were present at frequencies below the limit of reliable analysis in our model. Investigating of metabolic inhibition on Th17 responses will require models with more prominent IL-17-driven immunopathology. Importantly, DON compromises the establishment of virus-specific polyfunctional T-cell priming, which subtly impairs viral control and the immune memory for re-infection [33]. However, it preserves a baseline level of monofunctional activity in both CD4 + and CD8+ T cells. This residual immunity sustains essential immune surveillance, accounting for the observed tissue protection without viral explosion. While the overall memory T cell pool is diminished by DON, we observed a relative predominance of TCM within this pool. This is because TCM primarily rely on OXPHOS for their persistence, a metabolic pathway less susceptible to DON compared to the robust protein and biosynthetic demands of TEM [51]. It should be noted that the immunosuppressive effect of DON is likely transient, owing to its short half-life and the continuous turnover of metabolic enzymes [52]. In a Sindbis virus infection model, Baxter et al. reported that DON suppressed virus-specific IgM and IgG antibody levels, which recovered after treatment cessation [33]. This indicates that the metabolic inhibition is transient and reversible, and does not cause permanent impairment to the capacity for mounting normal immune responses.

Notably, although both 2DG and DON have been demonstrated to play critical roles in T cell activation and function [28,53], they exhibit distinct tissue protection profiles. The superior therapeutic efficacy of DON relative to 2DG originates from fundamental immunometabolic principles. Lymphocyte activation requires glutamine not only for energy generation but critically as an essential nitrogen donor for *de novo* nucleotide and amino acid biosynthesis [54,55]. This metabolic requirement underlies the significantly stronger suppression of T cell proliferation by DON compared to 2DG [47,48]. The susceptibility to glutamine deprivation creates a therapeutic window for acute self-limiting infection. This mechanistic insight aligns with clinical observations in COVID-19 and encephalomyelitis models, where metabolic interventions ameliorate cytokine storm without compromising host defense [41,56].

Our findings provide a mechanistic basis for glutaminase inhibitor application in CHIKV clinical management, wherein immunopathology suppression preserves muscle and joint integrity and potentially prevents CHIKV-induced chronic arthritis. A key limitation is that our model, using CHIKV strain KC488650 in adult mice, induces only an acute, self-limited infection with mild tissue injury without chronic arthritis pathology. This precludes an extended assessment of how DON affects adaptive immunological memory or recurrent arthritis, which limits direct clinical translation to chronic conditions. Moreover, regarding therapeutic potential historical gastrointestinal toxicity of DON remains a clinical application concern, next-generation glutaminase inhibitors (e.g. JHU083) are designed to be bio-activated specifically with minimizing systemic toxicity [57–59]. Importantly, upregulated glutaminolysis is a common metabolic feature of activated immune cells during pathogen-induced inflammation. The transient nature of glutaminase inhibitors supports their potential as a pulsed intervention during peak immunopathology, balancing tissue protection against systemic immunosuppression risks. This timing-based, host-directed strategy may be applicable not only to CHIKV but also to other acute immunopathology-driven diseases, including those caused by related arboviruses. Future studies should evaluate pulsed regimens using next-generation inhibitors and their combination with antiviral agents in diverse models that vary by viral strain, host age, sex, and infection course (acute vs. chronic), to optimize their effects on viral clearance, persistence and long-term protection.

## Supplementary Material

supplementary_flies_revised_20260104-clean.docx

## Data Availability

The datasets generated and/or analyzed during the current study are available from the corresponding author on reasonable request.
