# Supplementary material for: Glutamine antagonist DON attenuates chikungunya virus-induced myositis by suppressing inflammatory activation in a murine model
Source: Emerg Microbes Infect. 2026 Mar 6;15(1):2622213. doi: 10.1080/22221751.2026.2622213 (PMC12973807; doi:10.1080/22221751.2026.2622213)
Supplement: supplementary_flies_revised_20260104-clean.docx [file TEMI_A_2622213_SM9323.docx]

**Glutamine Antagonist DON Attenuates Chikungunya Virus-Induced Myositis by Suppressing Inflammatory Activation in a Murine Model**

**Supplementary Figure Legends**


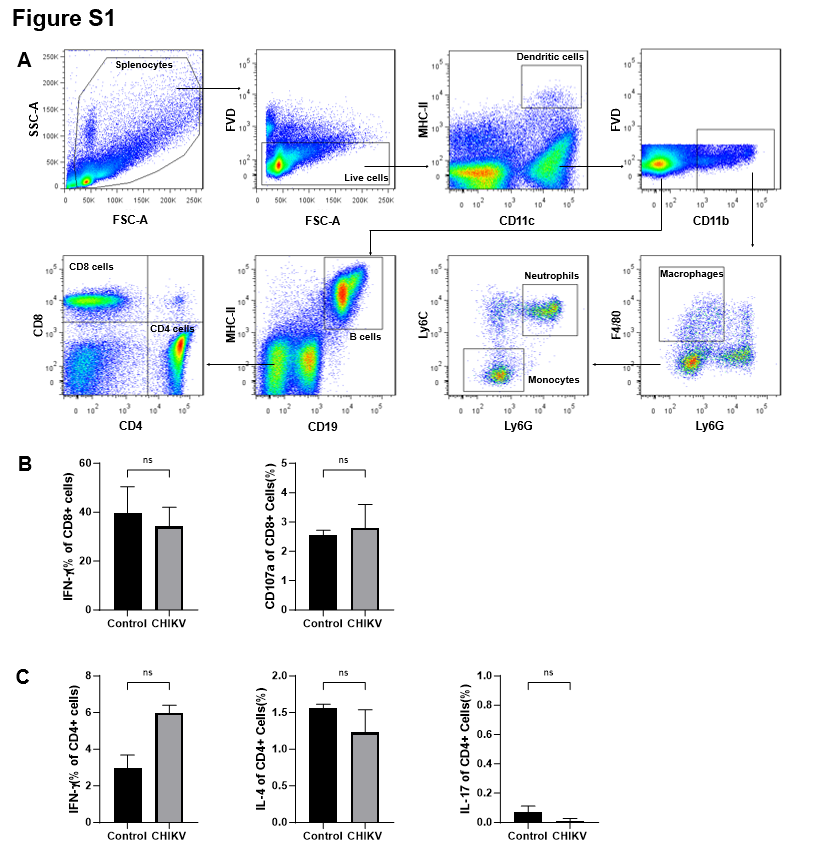


**Figure S1. Gating strategy for splenic immune cell subsets.** A. Live cells (FVD-) were analyzed for: DCs (CD11c+MHC-II+), macrophages (CD11b+F4/80+), monocytes (CD11b+Ly6G-Ly6C-), neutrophils (CD11b+Ly6G+Ly6C+), B cells (CD19+MHC-II+), CD4+ T cells (CD4+CD8-) and CD8+ T cells (CD4-CD8+). B. IFN-γ or CD107a producing CD8+ T cells, and (C) Th1 (IFN-γ+), Th2 (IL-4+) or Th17 (IL-17+) CD4+ T cells were assessed by flow cytometry at 16 dpi following 4.5 h stimulation with PMA/Ionomycin.


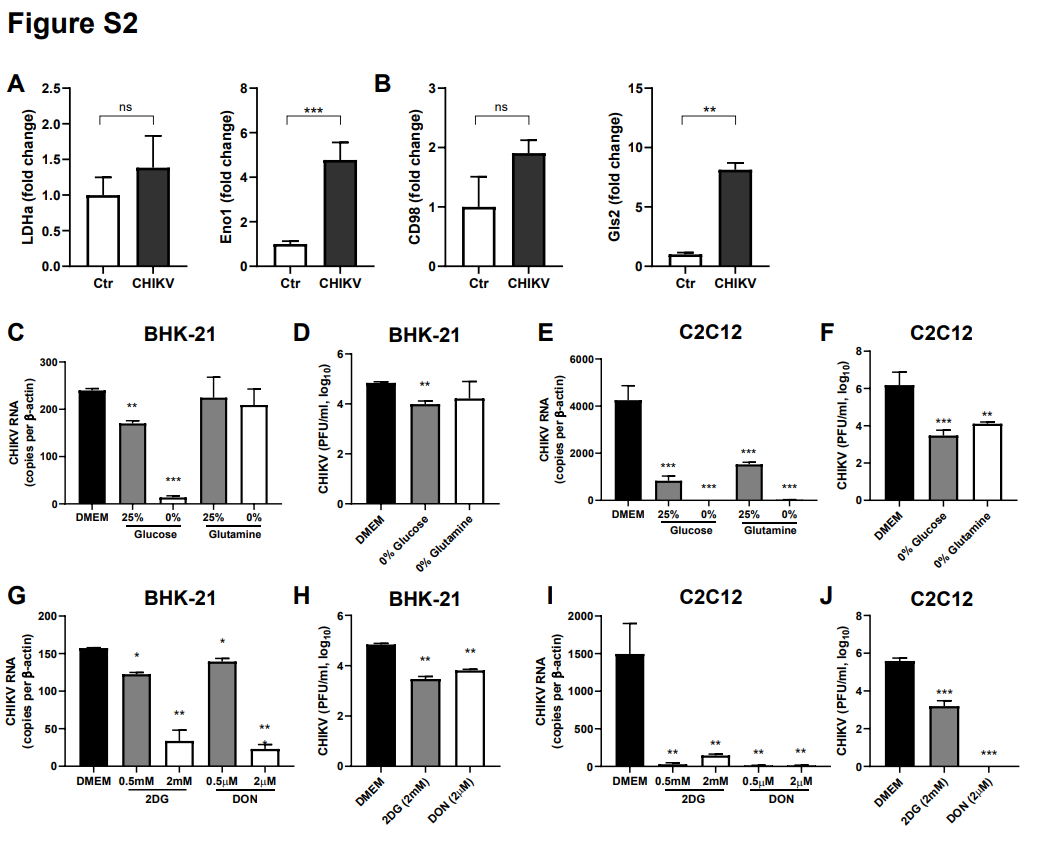


**Figure S2. Metabolic dependence on glucose and glutamine during CHIKV infection *in vitro*.** BHK21 and C2C12 cells were infected with CHIKV at MOI=1. Uninfected BHK21 cells served as controls (Ctr). (A) Glycolysis-related genes (*LDHa*, *Eno1*) and (B) glutaminolysis-related genes (*CD98*, *Gls2*) expression were detected by qRT-PCR. (C, D) Viral replication in BHK21 cells or (E, F) C2C12 cells under glucose or glutamine deprivation were assessed by qRT-PCR or plaque assay. (G, H) Viral replication in BHK21 cells or (I, J) C2C12 cells following 2DG or DON treatment were assessed by qRT-PCR or plaque assay.
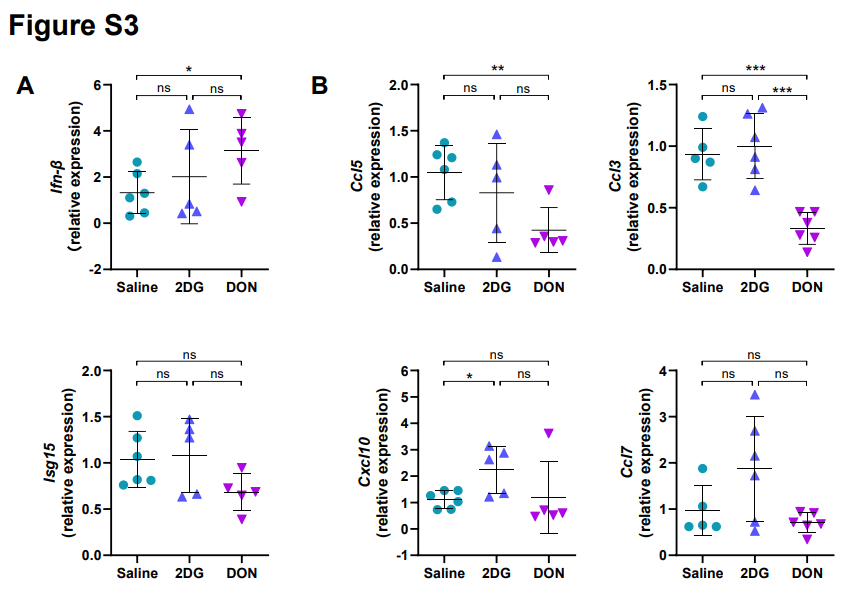


**Figure S3. Impact of metabolic inhibitors on innate immune responses in foot tissue.** C57BL/6 mice received a single intraperitoneal injection of 2DG (1 g/kg), DON (0.3 mg/kg) or saline. Foot tissues were harvested at 24 h post-treatment. (A) mRNA expression levels of antiviral interferon-stimulated genes (*Ifnb*, *Isg15*) and (B) inflammatory chemokines (*Ccl5*, *Ccl3*, *Cxcl10*, *Ccl7*) were quantified by qRT-PCR. Relative gene expression was normalized to β-actin and calculated relative to saline treated controls.. Data represented mean ± SD (n=5-6 mice per group). Statistical significance was determined by one-way ANOVA. ns: not significant; *: *p* < 0.05; **: *p* < 0.01; ***: *p* < 0.001.

**Table S1. Detailed list of key resources.** Summary of metabolic inhibitors, cell culture reagents, flow cytometry antibodies (including clones and fluorochromes), commercial kits, stimuli, and instruments used in the experiments. Source information and catalog identifiers are provided for each item.

| **Category** | **Reagent/Resource Name** | **Clone / Equipment** | **Manufacturer** | **Catalog Number** |
| --- | --- | --- | --- | --- |
| Metabolic Inhibitors | 2-Deoxy-D-glucose (2-DG) | N/A | Sigma-Aldrich | D8375 |
|  | 6-Diazo-5-oxo-L-norleucine (DON) | N/A | Sigma-Aldrich | D2141 |
|  | Oligomycin | N/A | Sigma-Aldrich | O4876 |
|  | Puromycin | N/A | Sigma-Aldrich | P8833 |
| Cell Culture | DMEM (High Glucose) | N/A | Gibco | 11965092 |
|  | Glucose-free DMEM | N/A | Gibco | 11966025 |
|  | Glutamine-free DMEM | N/A | Gibco | 11960044 |
|  | Fetal Bovine Serum (FBS) | N/A | Gibco | 10099141 |
|  | Dialyzed FBS | N/A | Gibco | A3382001 |
|  | Penicillin-Streptomycin | N/A | Gibco | 15140122 |
|  | 1.25% Methyl Cellulose Medium | N/A | Sigma-Aldrich | M0512 |
| Flow Cytometry Abs | Anti-mouse CD4 | GK1.5 | BioLegend | 100438 |
|  | Anti-mouse CD8a | 53-6.7 | BioLegend | 100712 |
|  | Anti-mouse CD11b | M1/70 | BioLegend | 101216 |
|  | Anti-mouse CD11c | N418 | BioLegend | 117318 |
|  | Anti-mouse CD19 | 6D5 | BioLegend | 115520 |
|  | Anti-mouse CD44 | IM7 | BioLegend | 103028 |
|  | Anti-mouse CD45 | 30-F11 | BioLegend | 103116 |
|  | Anti-mouse CD62L | MEL-14 | BioLegend | 104418 |
|  | Anti-mouse CD107a | 1D4B | BioLegend | 121614 |
|  | Anti-mouse CD127 | A7R34 | BioLegend | 135014 |
|  | Anti-mouse F4/80 | BM8 | BioLegend | 123116 |
|  | Anti-mouse Ly6C | HK1.4 | BioLegend | 128016 |
|  | Anti-mouse Ly6G | 1A8 | BioLegend | 127618 |
|  | Anti-mouse MHC-II | M5/114.15.2 | BioLegend | 107626 |
|  | Anti-mouse IFN-γ | XMG1.2 | BioLegend | 505808 |
|  | Anti-mouse TNF-α | MP6-XT22 | BioLegend | 506308 |
|  | Anti-mouse IL-17A | TC11-18H10.1 | BioLegend | 506916 |
|  | Anti-mouse Ki-67 | 16A8 | BioLegend | 652404 |
|  | Anti-mouse Granzyme B | QA16A02 | BioLegend | 372208 |
|  | Anti-mouse CXCR3 | CXCR3-173 | BioLegend | 126514 |
|  | Anti-Puromycin | 2A4 | BioLegend | 381508 |
|  | Fixable Viability Dye eFluor 780 | N/A | eBioscience | 65-0865-14 |
| Kits | Cytofix/Cytoperm Kit | N/A | BD Biosciences | 554714 |
|  | Foxp3/Transcription Factor Staining Set | N/A | eBioscience | 00-5523-00 |
|  | Mouse IFN-γ ELISPOT Kit | N/A | eBioscience | 88-7384-88 |
| Stimulants | PMA (Phorbol 12-myristate 13-acetate) | N/A | Sigma-Aldrich | P1585 |
|  | Ionomycin | N/A | Sigma-Aldrich | I0634 |
|  | GolgiStop (Monensin) | N/A | BD Biosciences | 554724 |
|  | Pam3CSK4(P3C) | N/A | InvivoGen | 112208-01-2 |
| Instruments | Tissue Cell Destroyer | D1000 | NZK Ltd. (Wuhan) | N/A |
|  | Flow Cytometer | LSRFortessa | BD Biosciences | N/A |
|  | ABSL-3 Isolators | Isocage | Tecniplast (Italy) | N/A |
|  | Digital Caliper | General | Mitutoyo/Generic | N/A |
| Experimental Models | C57BL/6 Mice | N/A | Beijing Vital River | N/A |
|  | BHK-21 Cells | N/A | ATCC | CCL-10 |
|  | C2C12 Cells | N/A | ATCC | CRL-1772 |
|  | CHIKV Strain | KC488650 | WIV, CAS | N/A |
